# Statin Use and Its Impact on Survival in Pancreatic Cancer Patients

**DOI:** 10.1097/MD.0000000000003607

**Published:** 2016-05-13

**Authors:** Hee Seung Lee, Sang Hoon Lee, Hyun Jik Lee, Moon Jae Chung, Jeong Youp Park, Seung Woo Park, Si Young Song, Seungmin Bang

**Affiliations:** From the Department of Internal Medicine, Institute of Gastroenterology, Yonsei University College of Medicine, Seoul, Korea.

## Abstract

Statins are cholesterol-lowering medications that are associated with a number of signaling pathways involved in carcinogenesis. Recent observational studies raised the possibility that the use of statins may reduce overall mortality in various types of cancer. We investigated whether statins used after pancreatic cancer diagnosis are associated with longer survival in pancreatic cancer patients.

We retrospectively analyzed data from 1761 patients newly diagnosed with pancreatic adenocarcinoma between January 1, 2006, and December 31, 2014. We used the time-dependent Cox proportional hazards regression model to estimate mortality among pancreatic cancer patients according to statin use.

Among the 1761 pancreatic cancer patients, 118 patients had used statins. During the study period, 1176 patients (66.7%) died. After adjusting for age, sex, location of cancer, cancer stage, diabetes mellitus, hypertension, dyslipidemia, smoking, alcohol use, body mass index, and CA 19-9, statin use was associated with a lower risk of cancer death (hazard ratio [HR], 0.780; 95% confidence interval [CI], 0.617–0.986), especially among simvastatin users (HR, 0.554; 95% CI, 0.312–0.982) and atorvastatin users (HR, 0.636; 95% CI, 0.437–0.927). Subgroup analysis showed that overall survival was statistically significantly longer in patients with nonmetastatic pancreatic cancer (log-rank *P* = 0.024).

We found that the use of simvastatin and atorvastatin after cancer diagnosis is associated with longer survival in patients with nonmetastatic pancreatic adenocarcinoma.

## Abstract 2

Supplemental Digital Content is available in the text

## INTRODUCTION

The prognosis for patients with pancreatic cancer remains poor, with an estimated 5-year survival rate of 6%.^1^ Although various anticancer agents have been developed over the years, no effective treatments for this lethal cancer have reached the clinic level to date.

3-hydroxy-3-methylglutaryl coenzyme A (HMG-CoA) reductase inhibitors, known as statins, have been shown to improve lipid profiles and to reduce cardiovascular morbidity and mortality through therapeutic and preventative effects in coronary artery diseases.^2^ Because of their pleiotropic effects, they have been of considerable interest for cancer prevention and treatment. Statins have anticancer effects through the inhibition of post-translational modification of key proteins involved in tumor proliferation and metastasis through the mevalonate pathway.^3^ To date, a number of observational studies have shown an inverse association between statin use and overall survival in patients with different types of cancers.^4–7^

The results from previous studies of statin use and overall survival in pancreatic cancer patients are inconsistent.^8,9^ Recently, Jeon et al showed that statin treatment is associated with enhanced survival in patients with pancreatic cancer.^10^ However, the analysis was limited to elderly patients >65 years of age who have more comorbid conditions and a shorter survival rate than younger populations. Moreover, they did not report on the differences in overall survival according to the duration of statin use; this information is necessary for the development of recommendations regarding the therapeutic use of statins in these populations.

To further examine the association between statin use and overall survival in pancreatic cancer patients, we analyzed data collected in a tertiary referral hospital in the Republic of Korea. As such, we aimed to determine whether the use of statins after cancer diagnosis is associated with a longer survival in patients with pancreatic adenocarcinoma.

## METHODS

### Study Population

This study was conducted by obtaining patient data from the Yonsei Tumor Registry database at Severance Hospital. The Yonsei Tumor Registry contains tumor information, such as tumor grade, clinical stage, pathological stage, treatment, cancer location, pathology, operation name, and diagnostic method. As the first step, we retrospectively analyzed the medical records of patients newly diagnosed with pancreatic cancer between January 1, 2006, and December 31, 2014. Cancer diagnosis was classified according to the International Classification of Diseases, 10th Revision (ICD-10) codes. We excluded patients (1) who were aged < 20 years at diagnosis, (2) who were aged ≥ 90 years at diagnosis, (3) who were diagnosed with pancreatic cystic neoplasm, intraductal papillary mucinous neoplasm (IPMN), neuroendocrine carcinoma, lymphoma, or (4) whose diagnosis was not confirmed by pathological review. This study conformed with the ethical guidelines of the Declaration of Helsinki (1975), and it was approved by the independent institutional review board of Severance Hospital, Yonsei University College of Medicine, Seoul, Republic of Korea.

### Variables

This study collected data regarding age at diagnosis, sex, body mass index (BMI), weight, height, diabetes mellitus (DM), hypertension (HTN), chronic pancreatitis, cholesterol level at diagnosis, family history of pancreatic cancer, smoking history, alcohol use, carbohydrate antigen (CA) 19-9 level at diagnosis, cancer location, cancer stage at diagnosis, histologic grade, and the type of treatment the patient received. Ages at the time of pancreatic cancer diagnosis were grouped as follows: < 50, 51 to 70, and ≥ 70 years. BMI was computed as weight/height^2^ (kg/m^2^) and grouped according to World Health Organization categories. Smoking and alcohol history were divided into 2 groups: nonsmoker (or nondrinker), former or current smoker (or drinker). The 7th edition of the tumor-node-metastasis system from the American Joint Committee on Cancer was used to determine the clinical stage of the study patients.^11^ In inoperable patients, clinical cancer stage was confirmed by imaging studies, including ultrasonography, computed tomography, magnetic resonance imaging, and positron emission tomography. Pathological cancer stage was confirmed based on postoperative pathological results. Overall survival (OS) was defined as the interval from the diagnosis date to the date of death from any cause or last follow-up.^12^

### Statin Exposure Assessment

Statin users were defined as patients who received statin medications at least 30 days after pancreatic cancer diagnosis. To minimize the potential effect of reverse causation, we excluded patients who received statins before pancreatic cancer diagnosis, even if they continued the medication after diagnosis. We reviewed the drug name, the date of dispensing, the cumulative duration of statin use, and the cumulative dose of statin intake.

The statins prescribed during the study period were simvastatin, atorvastatin, rosuvastatin, pravastatin, and fluvastatin. Those statins can be classified by their pharmacologic properties: high potency statins (atorvastatin, rosuvastatin, simvastatin) or low potency statins (fluvastatin, pravastatin); lipophilic statins (atorvastatin, fluvastatin, simvastatin) or hydrophilic statins (pravastatin, rosuvastatin); and natural statins (simvastatin, pravastatin) or synthetic statins (atorvastatin, rosuvastatin, fluvastatin). The brand names of statins used in this study were as follows: simvastatin (Cholesnone, Simvastar, Vytorin); atorvastatin (Atorva, LipiLOU, Lipitor); rosuvastatin (Crestor, Vivacor); pravastatin (Mevalotin); and fluvastatin (Lescol-XL).

The cumulative duration of statin use was classified into the following 3 categories: <6 months, 6 to 12 months, and >12 months. The cumulative dose was standardized for different statins using the defined daily doses (DDDs) recommended by the World Health Organization.^13,14^ The DDD for the 30 mg formulation of simvastatin was used as a reference, and the DDDs for the other statins were used to convert each statin dose to a dose equivalent to 30 mg of simvastatin. This variable was classified into the following 3 categories: <180 mg, 180 to 365 mg, and >365 mg of the DDD.

### Statistical Analysis

Data are expressed as a median, n (%), as appropriate. In order to evaluate statistically significant differences in baseline characteristics between statin users and nonusers, variables were compared using the chi-squared test for categorical data and Student's *t*-test for continuous variables. We estimated the median overall survival from diagnosis to death according to statin use by using Kaplan–Meier curves, and we compared these values using the log-rank test. Time-dependent Cox proportional hazards models were used to estimate hazards ratios (HRs) with 95% CIs of pancreatic cancer mortality associated with the postdiagnostic use of statins, cumulative duration of use, and cumulative dose of statins.

We evaluated the independent association of statin use after cancer diagnosis with overall survival by sequentially adding the following potential confounders: age at diagnosis, sex, cancer stage and location, BMI, DM, HTN, cholesterol, CA 19-9 at diagnosis, alcohol use, and smoking history. A *P*-value of < 0.05 was considered to be statistically significant. Statistical analyses were performed using SPSS version 18.0 (SPSS, Chicago, IL).

## RESULTS

### Patient Characteristics

**FIGURE 1:**
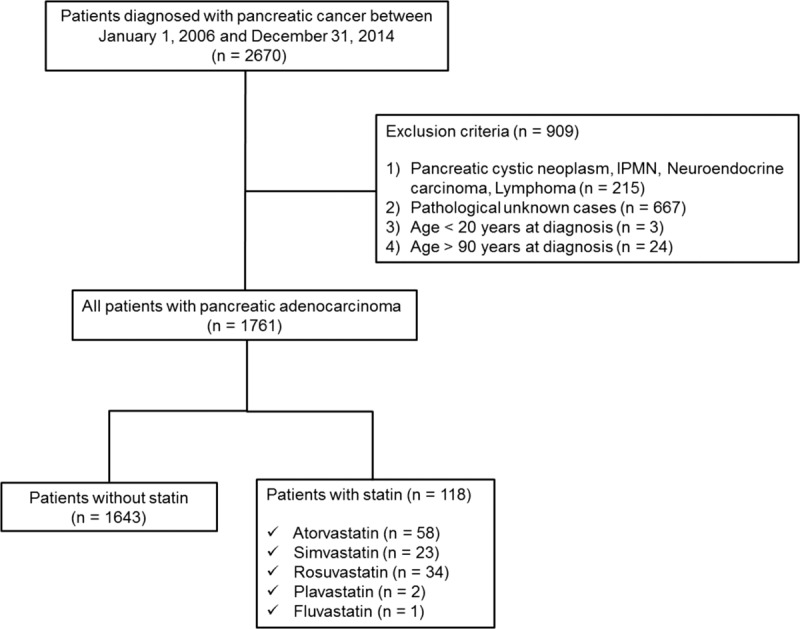
Study flowchart.

A total of 1761 patients newly diagnosed with pancreatic adenocarcinoma were included in this study (Figure 1). The baseline characteristics of the patients in this study are shown in Table 1. The mean age of the study population (which included 1035 males and 726 females) was 62.5 years. In this study, a total of 1183 (67.1%) patients received palliative chemotherapy. Regarding 1st line chemotherapy, 953 patients received gemcitabine-based chemotherapy and 230 patients received 5-fluorouracil-based chemotherapy. Of those, 417 patients received >4 cycles and 168 patients received >8 cycles of chemotherapy. Statin use after pancreatic cancer diagnosis was reported in 118 (6.7%) of the patient population. Atorvastatin was most commonly prescribed (49.2%), followed by rosuvastatin (28.9%) and simvastatin (19.5%), as described in Table 2. Among patients that received statins during the study period, the median average daily dose was 21 mg/day, and the median duration of use was 3.5 months. In the entire cohort, 585 (33.3%) patients survived until the last follow-up, 1176 (66.7%) patients died from any cause, and 1164 (66.0%) patients died of pancreatic cancer (Supplementary Table 1).

**TABLE 1:**
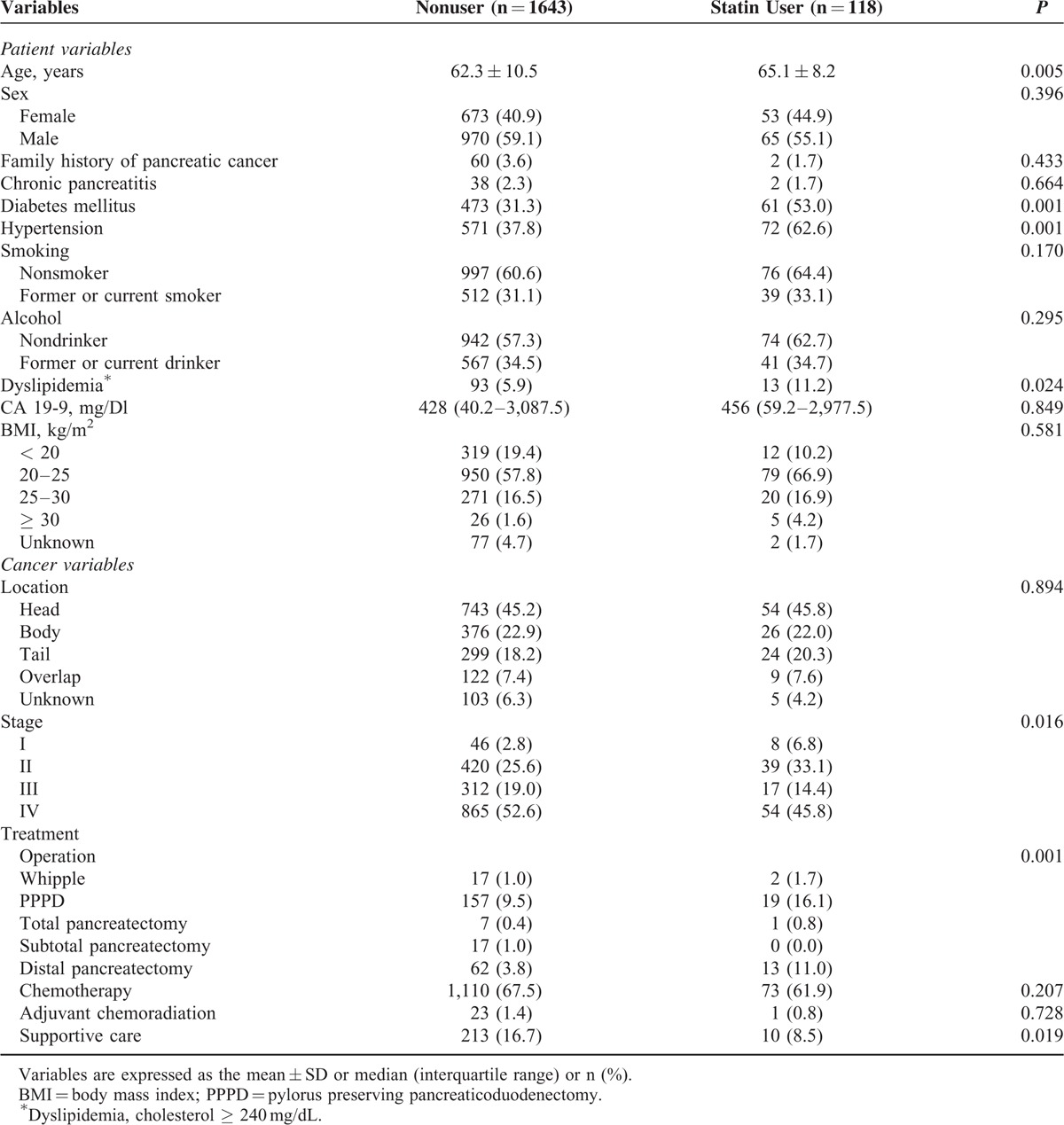
Baseline Characteristics of Study Participants (n = 1761)

**TABLE 2:**
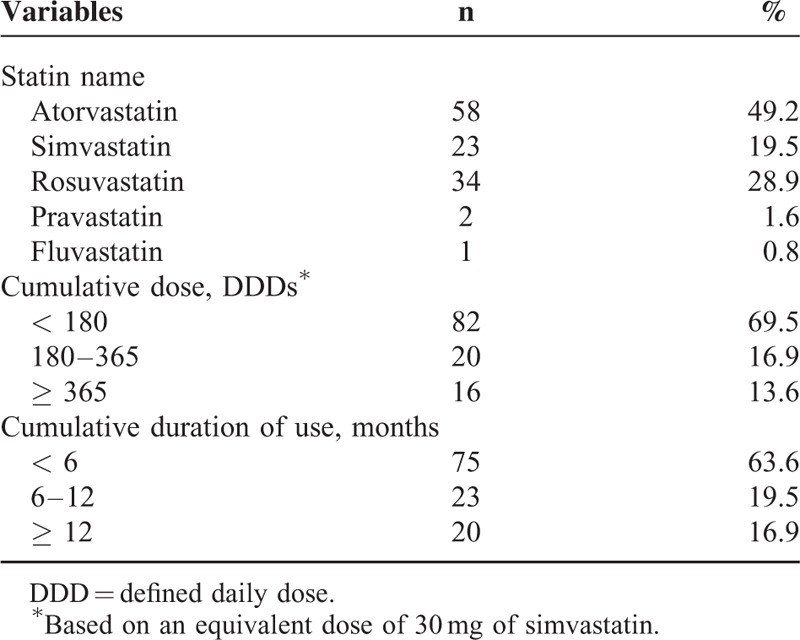
Baseline Characteristics of Statin Use (n = 118)

### Statin Use and Survival

**FIGURE 2:**
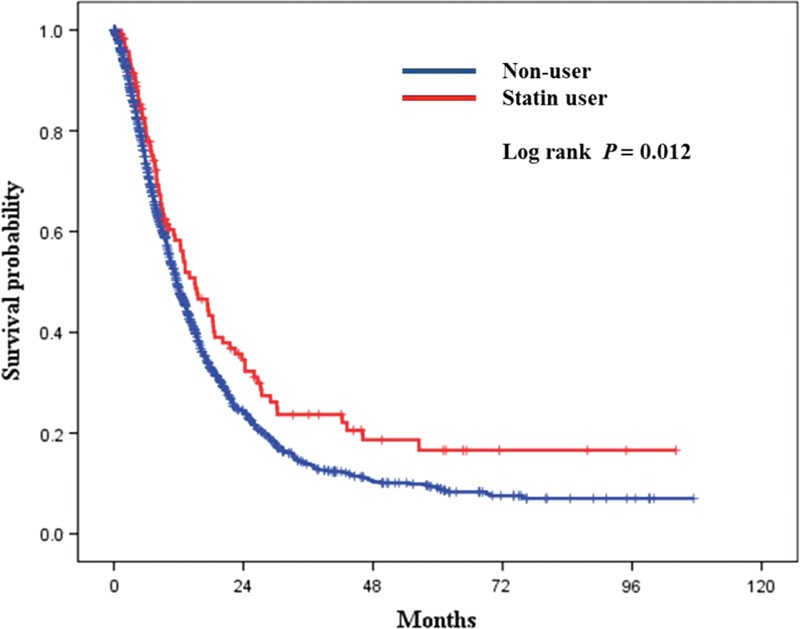
Survival analysis according to statin therapy. Kaplan–Meier curves for overall survival in pancreatic cancer patients with statin use and without statin use. (Log-rank *P* = 0.012, 5-year overall survival: nonstatin group—8.9%, statin group—16.6%).

**FIGURE 3:**
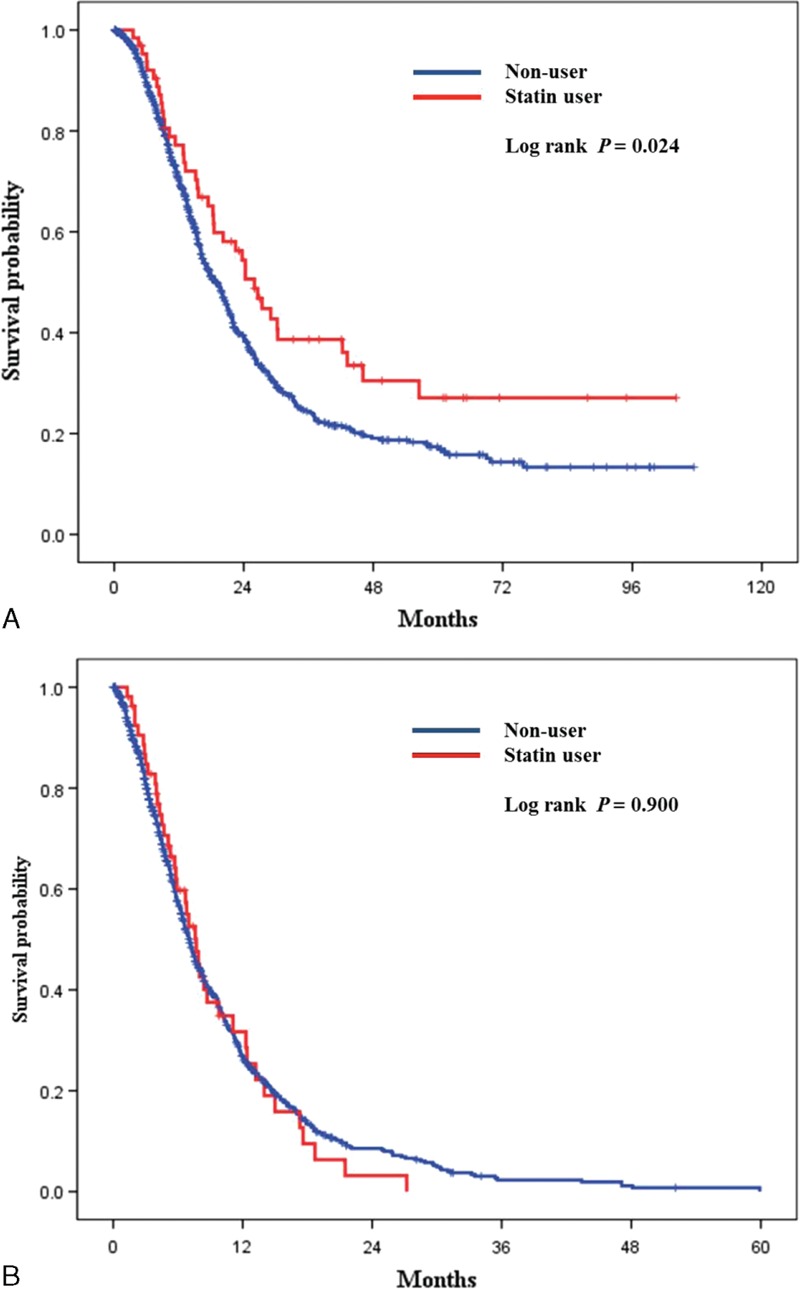
Subgroup analysis according to cancer stage. (A) Kaplan–Meier curves for overall survival in nonmetastatic pancreatic cancer patients (stages I, II, III). (Log-rank *P* = 0.024, nonuser [n = 778], Statin user [n = 64]). (B) Kaplan–Meier curves for overall survival in metastatic pancreatic cancer patients (stage IV). (Log-rank *P* = 0.900, nonuser [n = 865], Statin user [n = 54]).

We compared the survival outcomes of statin users (*N* = 118) and nonusers (*N* = 1643) (Figure 2). Statin users had significantly longer overall survival compared to nonusers (*P* = 0.012 by the log-rank test). Five-year overall survival was 16.6% for statin users and 8.9% for nonusers. We stratified the patients according to their cancer stage to show the effect of statins in patients according to cancer stage (stage I, II, III, *N* = 64 vs IV, *N* = 54). We compared the survival outcomes of statin users and nonusers (Figure 3). On subgroup analysis, statin users had significantly longer overall survival compared to nonusers in nonmetastatic pancreatic cancer (*P* = 0.024 by the log-rank test).

### Factors Associated With Overall Survival

We used multivariate analysis to assess the factors associated with survival in pancreatic cancer patients (Table 3). According to the multivariate analysis, cancer stage (HR = 5.058; 95% CI, 3.474–7.363), hypertension (HR = 1.182; 95% CI, 1.040–1.343), sex (HR = 1.178; 95% CI, 1.041–1.332), and age at diagnosis (HR = 1.456; 95% CI, 1.157–1.833) were identified as independent factors associated with survival. The use of statins after pancreatic cancer diagnosis was associated with decreased pancreatic cancer mortality after adjusting for all potential confounders (HR = 0.780; 95% CI, 0.617–0.986). Use of statins after pancreatic cancer diagnosis was associated with a 22% risk reduction in pancreatic cancer mortality (Table 3).

**TABLE 3:**
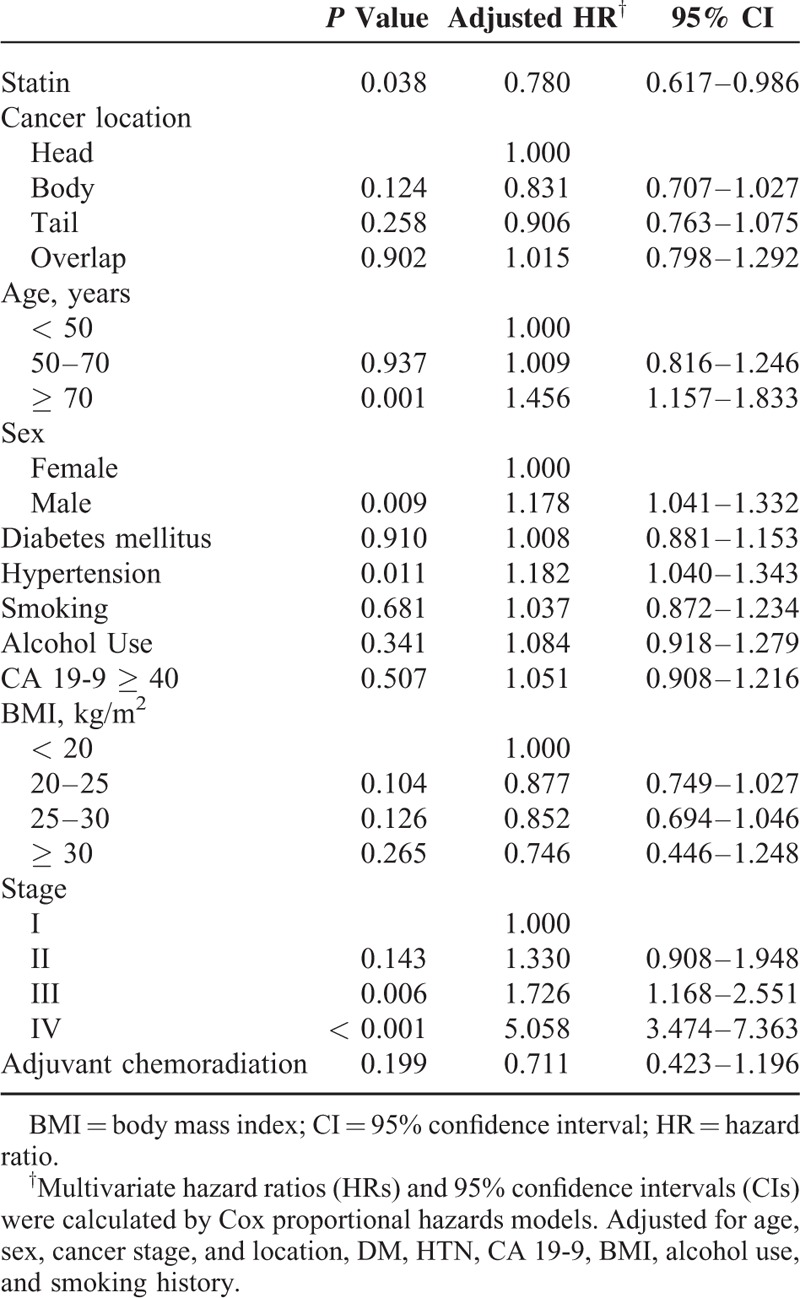
Multivariate Analysis of Risk Factors Associated With Overall Survival

### Cumulative Duration and Dose of Statin Use

We used the time-dependent analysis to control the immortal time bias because patients who live longer could have higher chance to use statins and higher chance to have longer prescription duration. On analysis, cumulative duration of statin use was associated with survival in patients. A duration response relationship was observed, with the HR decreasing in association with longer durations of statin use compared with nonstatin use (Table 4). The patients who used statins for >6 months after diagnosis received the benefits compared with those who did not use statins (≥ 6 months use: HR = 0.535; 95% CI, 0.315–0.911). When statin use was categorized by cumulative dose, the adjusted HRs were 0.576 (95% CI, 0.354–0.937) for the group with cumulative statin use of 180 DDDs or more compared with nonusers.

**TABLE 4:**
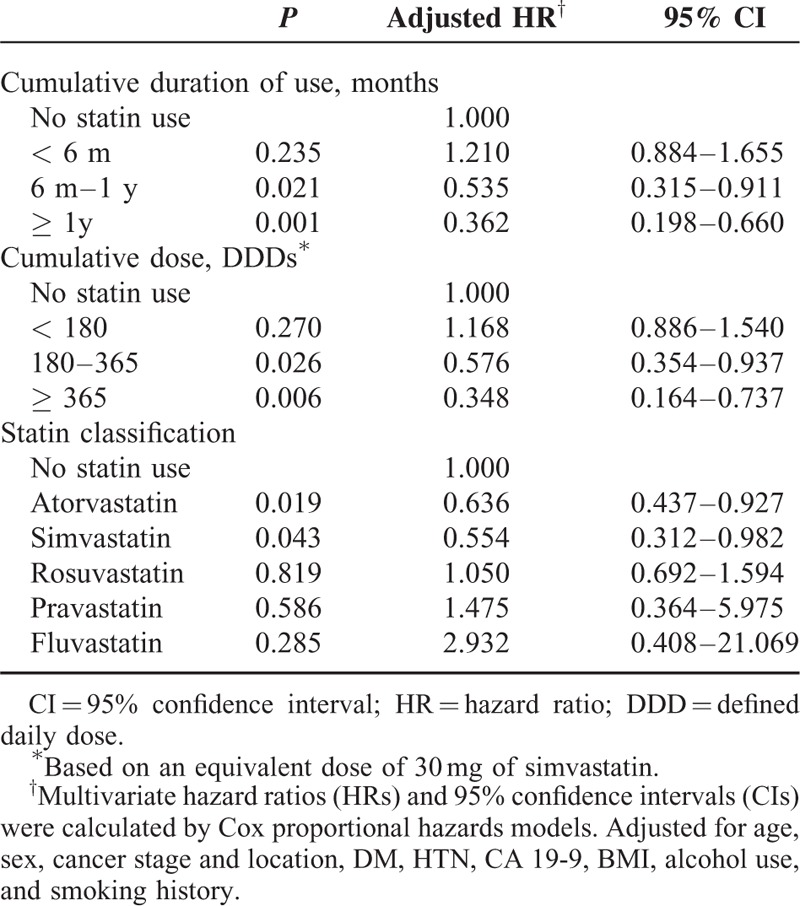
The Effect of Statin Use on Overall Survival According to Duration of Statin Use and Type of Statin

### Statin Classification

Analysis by statin type revealed statistically significant inverse associations for the 2 commonly prescribed statins: simvastatin (adjusted HR = 0.554; 95% CI, 0.312–0.982) and atorvastatin (adjusted HR = 0.636; 95% CI, 0.437–0.927). Patients who received simvastatin and atorvastatin were likely to show a survival benefit compared to nonusers (Table 4).

## DISCUSSION

The results of this large, population-based study indicate that the postdiagnostic use of statins, particularly use of simvastatin and atorvastatin, is associated with longer survival in patients with nonmetastatic pancreatic adenocarcinoma.

Antitumor effects by statin have been demonstrated in various experimental studies.^15–21^ Cancer cells are fast-growing cells that are characterized by increased glycolysis and lipid biosynthesis to meet the metabolic needs of the cells and to provide cholesterol for cell membrane stability and growth, which can ultimately lead to angiogenesis.^22^ The main effect of statins on cancer cells is the inhibition of cholesterol synthesis through the inhibition of HMG-CoA reductase, the rate-limiting enzyme in the cholesterol-synthesis pathway,^23^ which may lead to increased cancer cell apoptosis.^24^

Statins also inhibit the formation of farnesyl pyrophosphate (FFP) and geranylgeranyl pyrophosphate (GGPP) in the mevalonate pathway when they inhibit HMG-CoA reductase. FPP and GGPP bind to Ras proteins (small GTPase related to cellular signal transduction) through an isoprenylation process. Therefore, Ras proteins are not able to be active by inhibiting the formation of FPP and GGPP with statins, which ultimately result in apoptosis of the cancer cells.^22,25^

To date, a number of observational studies have examined the association between statins and pancreatic cancer outcomes, with mixed finding.^10,26–28^ With respect to pancreatic cancer mortality, 1 study reported that statins were associated with a longer survival in pancreatic cancer, whereas another study observed no statistically significant reduction in mortality.^10,27^ Recently, Jeon et al showed that statin use after cancer diagnosis was associated with a 21% reduced hazard of death (HR = 0.79; 95% CI, 0.67–0.93) in patients with early stage pancreatic cancer.^10^ However, the study analysis was limited to elderly patients who have more comorbidities and who are less likely to have surgery compared to younger patients. In addition, there was no analysis regarding the association between mortality and the cumulative duration of statin use in pancreatic cancer patients. In another recent study, the authors showed that prior simvastatin use was associated with improved overall survival (28.5 vs 16.5 months, log rank *P* = 0.0035).^29^ However, the analysis was limited to statin use prior to surgery or prior to diagnosis in pancreatic cancer patients. The study did not evaluate effects on mortality when statins were initiated after diagnosis or surgery. In order to determine the most effective use of statins as an antitumor medication or to assess their effects on mortality, it is necessary to analyze how the cumulative use of statins and the dose of the statin taken after pancreatic cancer diagnosis affect outcomes in pancreatic cancer patients. To this end, we investigated the relationships between survival and history of statin usage, cumulative dosage, and the duration of statin use after diagnosis with pancreatic cancer.

In this study, there was a significant survival benefit associated with statin use after adjusting for confounding variables in statin users. The mortality reduction rate was comparable to rates found in previous studies.^10^ In similar studies of other types of cancer, we demonstrated the dose- and time-dependence of the survival benefit among statin users.^6,7^ These studies regarding the dose- and time-dependence of the survival benefit among statin users suggests that statins are a potential target for new cancer treatments.

Statins provided a significant survival benefit according to multivariate analysis adjusting for confounding factors, especially among simvastatin and atorvastatin users. There are a number of possible explanations for the observed survival effect related to postdiagnostic use of simvastatin and atorvastatin. A possible reason for the stronger effect of simvastatin is that simvastatin is a lipophilic statin. The difference between lipophilic and hydrophilic statins may be related to their different chemical structures, pharmacokinetic profiles. Lipophilic statins diffuse across cellular membranes and exert their metabolic effects. On the other hand, hydrophilic statins require active transport across the cell membrane in order to exert their actions intracellularly.^3,30^ Preclinical studies have also shown that lipophilic statins inhibit growth in breast cancer cell lines because of these characteristics.^31^

Tumor stage is the most important prognostic factor. In this study, the statins users are more in their early stage of pancreatic cancer. The survival benefit in statin users might be due to the cancer stage. Therefore, we stratified the patients to show the effect of statins in patients according to cancer stage. And, we found that statin users had significantly longer overall survival compared to nonusers in nonmetastatic pancreatic cancer.

This study has a number of strengths. To our knowledge, this is the largest study that has demonstrated an inverse association between statin use, cumulative duration, and survival after diagnosis in pancreatic cancer patients. In addition, this is the first study to demonstrate a significant association between statin use and pancreatic cancer mortality in an Asian population. This study also included all age groups and assessed the duration of statin use, the lack of which were weaknesses in previous studies.

We are also aware of several possible limitations to this study. First, this is a nonrandomized, retrospective study at a single center. The best study design to know whether statins could reduce the mortality rates of pancreatic cancer is prospective, randomized control trial, and not an observational study. Specifically, caution needs to be exercised with retrospective studies because errors are more common than in prospective studies due to confounders and bias. However, this limitation does not appear to have distorted our findings, as the large scale of this study has more power. With nearly 10 years of follow-up included in the study period, the study was well-powered to investigate the results of statin use on survival. Second, there may be a possible healthy-user bias, as statin users were more likely to be concerned with their health prior to cancer diagnosis than nonstatin users. However, it is unclear whether better lifestyle habits would fully explain the 22% risk reduction in pancreatic cancer mortality and the duration-response relationships observed with increased HR. Furthermore, statin users seemed to have a higher prevalence of comorbidities than nonstatin users, which is characteristically more likely to be associated with worse outcomes. However, statin users showed improved survival rates despite high comorbidities, which means that statins have an effect on survival. We also excluded patients who received statins before pancreatic cancer diagnosis, even if they continued the medication after diagnosis, to minimize the potential effect of reverse causation.

In conclusion, we found that Simvastatin and atorvastatin use after pancreatic cancer diagnosis is associated with longer survival in patients with nonmetastatic pancreatic adenocarcinoma. Finally, additional well-designed randomized controlled studies are needed to confirm these findings in the future.

## Supplementary Material

Supplemental Digital Content
