# Supplementary material for: Statin Use and Its Impact on Survival in Pancreatic Cancer Patients
Source: Medicine (Baltimore). 2016 May 13;95(19):e3607. doi: 10.1097/MD.0000000000003607 (PMC4902509; doi:10.1097/MD.0000000000003607)
Supplement: Supplemental Digital Content [file medi-95-e3607-s001.docx]

| **Supplementary Table 1. Reason for death in the patients with pancreatic cancer** | | | | | | |  |
| --- | --- | --- | --- | --- | --- | --- | --- |
| **Cause of death** |  | **Total patients**  **(n = 1,761)** |  | **Statin Users**  **(n = 118)** |  | **Non-Statin Users**  **(n = 1,643)** | |
| **All-cause death** |  | **1,176 (66.7%)** |  | **80 (67.8%)** |  | **1,096 (66.7%)** | |
| **Pancreatic cancer-specific death** |  | **1,164 (66.0 %)** |  | **78 (66.1%)** |  | **1,086 (66.1%)** | |
| **Others*** |  | **12 (0.7 %)** |  | **2 (1.7 %)** |  | **10 (0.6 %)** | |
| **Survival until last follow-up** |  | **585 (33.3 %)** |  | **38 (33.2 %)** |  | **547 (33.3 %)** | |

***Others include tongue cancer (n=1), colon cancer (n=2), lung cancer (n=3), duodenal cancer (n=1), esophageal cancer (n=1),**

**gastric cancer (n=1), stroke (n=1), gastrointestinal bleeding (n=1), and urinary tract infection (n=1)**
